# Phosphorylation of h1 Calponin by PKC epsilon may contribute to facilitate the contraction of uterine myometrium in mice during pregnancy and labor

**DOI:** 10.1186/1477-7827-10-37

**Published:** 2012-05-02

**Authors:** Lesai Li, Yong Zhang, Changju Zhou

**Affiliations:** 1 Department of Obstetrics and Gynecology, the third Xiangya Hospital, Central South University, Changsha, 410013, China; 2 Department of Hematology, Xiangya Hospital, Central South University, Changsha, 410008, China; 3 College of Medicine, Hunan Normal University, Changsha, Hunan, 410013, China

**Keywords:** h1 Calponin, PKC-epsilon, Phosphorylation, Mice, Labor, Pregnancy

## Abstract

**Background:**

The timely onset of powerful uterine contractions during parturition occurs through thick and thin filament interactions, similar to other smooth muscle tissues. Calponin is one of the thin filament proteins. Phosphorylation of calponin induced by PKC-epsilon can promote the contraction of vascular smooth muscle. While the mechanism by which calponin regulates the contraction of pregnant myometrium has rarely been explored. Here, we explore whether PKC-epsilon/h1 calponin pathway contribute to regulation of myometrial contractility and development of parturition.

**Methods:**

We detected the expression of h1 calponin, phosphorylated h1 calponin, PKC-epsilon and phosphorylated PKC-epsilon in the different stages of mice during pregnancy and in labor by the method of western blot and recorded the contraction activity of myometrium strips at the 19th day during pregnancy with different treatments by the organ bath experiments.

**Results:**

The level of the four proteins including h1 calponin, phosphorylated h1 calponin, PKC-epsilon and phosphorylated PKC-epsilon was significantly increased in pregnant mice myometrium as compared with that in nonpregnant mice. The ratios of phosphorylated h1 calponin/h1 calponin and phosphorylated PKC-epsilon/PKC-epsilon were reached the peak after the onset of labor in myometrium in the mice. After the treatment of more than 10(9-) mol/L Psi-RACK (PKC-epsilon activator), the contractility of myometrium strips from mice was reinforced and the level of phosphorylated h1 calponin increased at the same time which could be interrupted by the specific inhibitor of PKC-epsilon. Meanwhile, the change of the ratio of phosphorylated h1 calponin/h1 calponin was consistent with that of contraction force of mice myometrium strips.

**Conclusions:**

These data suggest that in mice myometrium, phosphorylation of h1 calponin induced by the PKC-epsilon might facilitate the contraction of uterine in labor and regulate pregnant myometrial contractility.

## Background

Labor may occur due to a loss of myometrial quiescence or an active increase in uterine contractility, or a combination of both. Several contractile-associated proteins have been proposed to contribute to reversal of quiescence and promote contraction of the uterus during labor [1,2]. Calponin is one of the thin filament proteins and a critical component of smooth muscle contractile machinery [3], while the mechanism by which calponin regulates the contraction of pregnant myometrium has rarely been explored. Calponin (CaP) was first isolated in smooth muscle over 20 years ago as a potential thin filament regulatory protein [4]. So far, Calponin is now known as a family of homologous actin filament-associated proteins expressed in both smooth muscle and non-muscle cells. Three isoforms of calponin have been found in the vertebrates as the products of three homologous genes: a basic calponin (smooth muscle-specific basic CaP, h1-calponin, h1 CaP) [5], a neutral calponin (h2-calponin) [6] and an acidic Calponin (h3 calponin) [7].

A series of animal experiments have been conducted about the role of calponin in smooth muscle contraction, the findings show the h1 calponin is specific to differentiated smooth muscle cells and up-regulated during post-natal development [8], which consistent with a role in contractile function. Calponin inhibits the contraction of smooth muscle through its interacting with actin and Calmodulin and inhibiting action-activated myosin ATPase activity without affecting myosin light chain (LC20) phosphorylation [9], but the phosphorylation of calponin induced by PKC, in particular PKC-ε displays a dramatic reduction in affinity for actin and is unable to inhibit myosin ATPase activity which promotes the contraction of smooth muscle. Phosphorylation of calponin by PKC-ε results in contraction of porcine coronary artery [10] whereas unphosphorylated calponin relaxes single permeabilized vascular smooth cells pre-contracted with phenylephrine and PKC-ε [11]. Previously, Winder [4] found Calponin was a substrate for PKC in 1990, followed by the discovery by Horowitz [11] that calponin is a substrate for PKC-ε. Whereafter, Menice [12] found that PKC-ε and h1 calponin co-immunoprecipitate and co-translocate to the vicinity of the plasmalemma in ferret vascular smooth muscle and that PKC-ε directly binds to h1 calponin in vitro [13]. The subcellular localization of calponin is also affected by PKC-dependent phosphorylation. Moreover, dephosphorylation of calponin by a type 2A protein phosphatase restores actin binding and inhibition of myosin ATPase [14]. In general, it has been postulated that agonist-induced, Ca2 + -independent contraction of smooth muscle is mediated by PKC-ε through its ability to phosphorylate calponin.

Although the function of h1 calponin in smooth muscle contraction has been extensively investigated, the physiological function of calponin in uterine myometrium is still inconclusive because relatively few studies have been performed in pregnant myometrium regarding the possible function of h1 calponin and PKC-ε. However, the information established from vascular smooth muscle studies laid a foundation for investigations on the function of h1 calponin in uterine myometrium. An increased protein level of h1 calponin in term pregnant human myometrium has been reported [15], but the mechanism by which h1 calponin regulates myometrium contraction has not been explored. Then, a new idea was presented whether the contraction of gestational myometrial was regulated by the PKC-ε/ h1 calponin signaling. Whether the inhibition of h1 calponin on actin may be reversed by PKC-ε-dependent phosphorylation in a gestation-related manner has not been explored. The purpose of the present study was to determine if the activation of PKC-ε and phosphorylation of h1 calponin might correlate with the onset of labor and whether phosphorylation of h1 calponin induced by PKC-ε regulates pregnant myometrial contractility in mice.

## Methods

### Animals and tissue handling

All procedures were approved by our institutional animal care and use committee. 8-week-old female BALB/C mice were randomly divided into seven groups (10 mice per group): nonpregnant(NP), pregnant D7(the 7th day during pregnancy), pregnant D12(the 12th day during pregnancy), pregnant D17(the 17th day during pregnancy), pregnant D18(the 18th day during pregnancy), pregnant D19(the 19th day during pregnancy), in labor (IL, after the first pup was delivered). Mice were killed by cervical dislocation. Uterine horns were excised and opened up along the mid-line. In pregnant mice, the placentae and fetuses were removed and pups decapitated. Longitudinal strips of myometrium were isolated from the tissue and freed of any endometrium, under a dissecting microscope, and cleaned of any blood by rinsing in Krebs solution (154 mM NaCl, 5.4 mM KCl, 1.2 mM MgSO4, 1.6 mM CaCl2, 5.5 mM glucose, and oxygenated with 95% O2-5% CO2, producing a pH of 7.4). The strips were snap frozen in liquid N2 until use, or prepared immediately for contractile studies.

### Western blot analysis

Muscles were stored in liquid N2 until processed as previously described for protein extraction for Western blotting [16]. The concentration of extracted proteins was determined by the Bradford Protein Assay kit (Santa Cruz, USA) according to the manufacturer’s instructions. Samples were subjected to electrophoresis on 10% SDS polyacrylamide gels. Gels were sealed with a blotting membrane. Membranes were placed into blocking buffer containing 5% non-fat milk and incubated for 2 hours at room temperature on a rocking platform. Primary antibody was added to each lane and incubated for 2 h, followed by three 10 min washes with TBST. Secondary antibody (1:10,000, Santa Cruz, USA) was then added to each lane and incubated for 1h, and the blot was subjected to three 10 min washes in TBST. Immunoreactivity was visualized by enhanced chemiluminescence (ECL Pierce Company, USA), images were captured on X-ray film (Kodak, American), and protein quantification was determined by using Gel Pro Analyzer 4 image analysis software (Media Cybernetics, USA). The h1 calponin antibody (1:3000, Millipore, USA) detected total h1 calponin protein levels. The phospho-h1 calponin antibody (1:1000, Santa Cruz, USA) used in this study was specific for the PKC (Protein kinase C) phosphorylation site at Ser175. The PKC-ε antibody (1:1000, Millipore, USA) was used to detect total PKC-ε protein levels. The phospho- PKC-ε antibody (1:1000, Santa Cruz, USA) used in this study detected phosphorylated PKC-ε which was catalytically activated by phosphorylation at Ser729.

### Contractile studies

Strips of myometrium (7 ~ 8 mm length × 2 ~ 3mm wide) were dissected from mice uterine at the time of Day 19 in pregnancy, tied at each end with silk thread and mounted vertically in 30-ml tissue baths. One tissue end was tied to a fixed hook, the other to an isometric tension transducer (Chengdu Instruments, China) and equilibrated in Krebs solution (154 mM NaCl, 5.4 mM KCl, 1.2 mM MgSO4, 1.6 mM CaCl2, 5.5 mM glucose, and oxygenated with 95% O2-5% CO2, producing a pH of 7.4). Experiments were performed at 37°C as previously described [17]. Preparations were allowed to equilibrate for 1 h before initiation of experiments. All myometrial strips were stretched to the optimal length, defined as the length corresponding to maximal force development with regard to spontaneous contraction [18]. The bath buffer solution was changed every 15 minutes during equilibration. The contractile activity was digitalized with BL-420E + biological and functional experimental system (Chengdu Technology & Market Co, LTD, China). To investigate the role of PKC-ε/ h1 calponin pathway to contractile activation, myometrial strips isolated from mice at the 19th day during pregnancy were stimulated with the PKC-ε specific activator (Psi-RACK, Anaspec, American), with the concentration ranging from 10^-12^ mol/L to 10^-6^ mol/L. The control group underwent a Psi-RACK concentration of 0 mol/L. To further investigate whether PKC-ε was involved in the phosphorylation of h1 calponin, 10^-9^ mol/L epsilon-V1-2 (PKC-ε specific inhibitor, Anaspec, American) was administered to the tissue baths after adding 10^-9^ mol/L Psi-RACK(PKC-ε activator) and not adding Psi-RACK groups. At the end of experiments, muscle strips were snapping frozen in liquid N2 for Western Blot Analysis.

### Statistical analysis

Data were presented as mean ± standard deviation (SD). Statistical significance was determined by one-way ANOVA using the SPSS 13.0 statistics package. Statistical significance was set at *P* < 0.05.

## Results

### The expression and phosphorylation of h1 calponin were increased during pregnancy and labor

**Figure 1:**
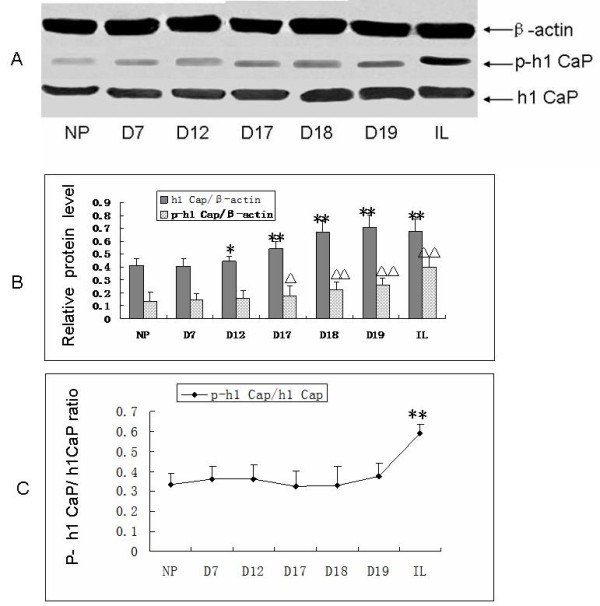
**The expression of h1 calponin and phosphorylated h1 calponin during different stages of pregnancy in mice.** Figure legend text Total protein extracted from the smooth muscle layer of mouse uterus was analyzed on SDS-PAGE and transferred to nitrocellulose membrane for western blotting with the anti-h1 calponin and anti-phosphorylated h1 calponin antibody. **A:** blots show the expression of h1 calponin and phosphorylated h1 calponin in the myometrium in different stages of pregnancy. **B:** columns from densitometry quantification of multiple western blots(means ± SD; n = 10) summarize the changes in the expression of h1 calponin and phosphorylated h1 calponin. **C:** curves from the ratio of phosphorylated h1 calponin to total h1 calponin. NP: non-pregnant; D7: Day 7 in pregnancy; D12: Day 12 in pregnancy; D17: Day 17 in pregnancy; D18: Day 18 in pregnancy; D19: Day 19 in pregnancy; IL: in labor; h1 Cap: h1 calponin; p- h1 Cap: phosphorylated h1 calponin .*: P < 0.05, compared with NP; **: P < 0.01, compared with NP; Δ: P < 0.05, compared with NP; ΔΔ: P < 0.01, compared with NP. Results demonstrated that total h1 calponin expression level gradually increased as the pregnancy progression, reached the peak before labor and continued until labor; the phosphorylation of h1 calponin expression levels increased gradually with the progress of pregnancy, reached the peak after labor. The ratio of phosphorylated h1 calponin to the total h1 calponin had no significant changes during pregnancy, but reached a peak after the onset of labor.

We explored the expression level of h1 calponin and phospho-h1 calponin in the samples of myometrium strips from nonpregnant mice or pregnant mice in different stages of pregnancy by the method of western blot with h1 calponin and phospho- h1 calponin (p- h1 calponin) antibodies. The phospho-h1 calponin antibody is specific for h1 calponin phosphorylated at a PKC phosphorylation site, Ser175. Band densities were quantitated by using Gel Pro Analyzer 4 image analysis software. The total h1 calponin protein levels were significantly increased in mice myometrium during pregnancy, in comparison with that in the strips from the nonpregnant mice (Figure 1A, 1B). However, the phospho-h1 calponin levels were significantly expressively increased in mice myometrium from the 17th Day during pregnancy till the labor in contrast with that in nonpregnant mice. Nevertheless, the ratio of phosphorylated h1 calponin/total h1 calponin was significantly increased in myometrium in the mice in labor as compared with that in pregnant or nonpregnant mice (Figure 1C). There was no significant difference in the ratio of phosphorylated h1 calponin/total h1 calponin among the nonpregnant mice and pregnant mice.

### The expression and phosphorylation of PKC-ε were increased during pregnancy and labor

**Figure 2:**
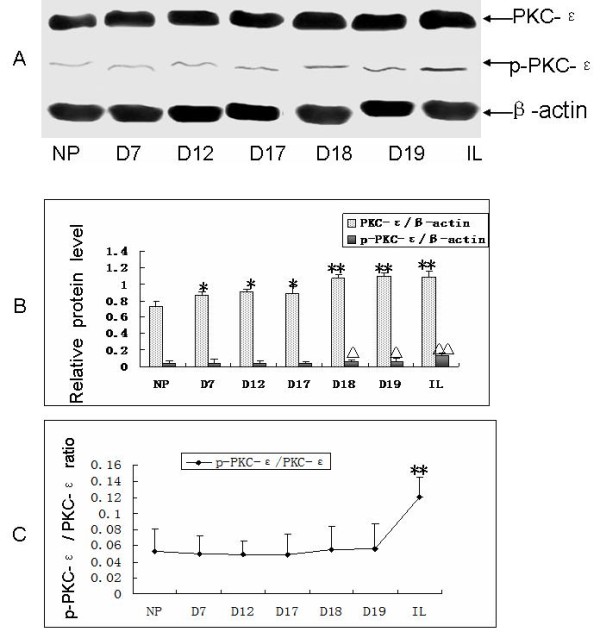
**The expression of PKC-ε and phosphorylated PKC-ε during different stages of pregnancy in mice. ** Total protein extracts from the smooth muscle layer of mouse uterus was analyzed on SDS-PAGE and transferred to nitrocellulose membrane for western blotting with the anti-PKC-ε and anti-phosphorylated PKC-ε antibody. **A:** blots show the expression of PKC-ε and phosphorylated PKC-ε in the myometrium in different stages of pregnancy. **B:** columns from densitometry quantification of multiple western blots (means ± SD; n = 10) summarize the changes in the expression of PKC-ε and phosphorylated PKC-ε. **C:** curves from the ratio of phosphorylated PKC-ε to total PKC-ε. NP: non-pregnant; D7: Day 7 in pregnancy; D12: Day 12 in pregnancy; D17: Day 17 in pregnancy; D18: Day 18 in pregnancy; D19: Day 19 in pregnancy; IL: in labor. *: P < 0.05, compared with NP; **: P < 0.01, compared with NP; Δ: P < 0.05, compared with NP; ΔΔ: P < 0.01, compared with NP. Results demonstrated that total PKC-ε expression level gradually increased as the pregnancy progression, reached the peak before labor and continued until labor; the phosphorylation of PKC-ε expression levels increased gradually with the progress of pregnancy, reached the peak after labor. The ratio of phosphorylated PKC-ε to the total PKC-ε had no significant changes during pregnancy, but reached a peak after the onset of labor.

We explored the expression level of PKC-ε and phospho-PKC-ε in the samples of myometrium strips from nonpregnant mice or pregnant mice in different stages of pregnancy by the method of western blot with PKC-ε and phospho-PKC-ε antibodies. Total PKC-ε protein level was significantly increased in pregnant or labor mice myometrium strips (Figure 2A, 2B) comparing with that in nonpregnant mice myometrium strips. The phospho-PKC-ε protein level got arising significantly from the 18th Day during the pregnancy till labor, contrasted with nonpregnant mice. But the ratio of phosphorylated PKC-ε/total PKC-ε was increased significantly in labor, which consistent with the time course of the changes in h1 calponin phosphorylation (Figure 2B, 2C).

### The effect of PKC-ε activator and inhibitor on the expression and phosphorylation of h1 calponin in the mice myometrium strips

**Figure 3:**
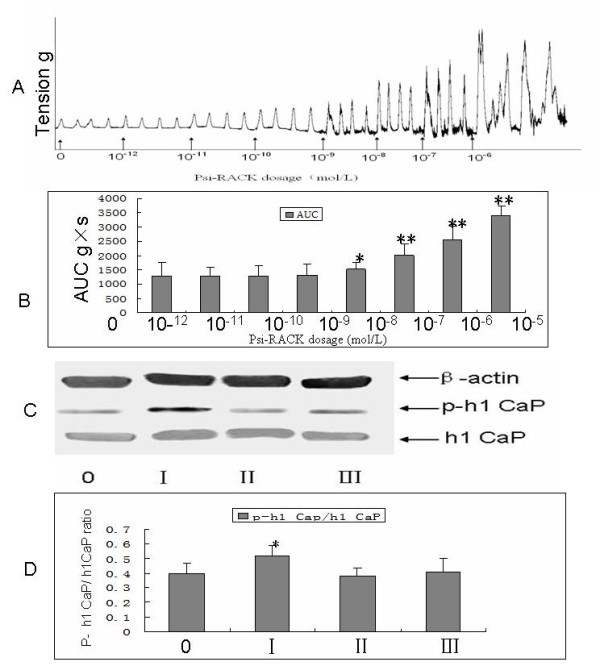
**The effect of PKC-ε specific activator (PSI-RACK) and inhibitor on the contraction of the mice myometrium strips at the day 19 during pregnant. A:** typical recording of the contraction amplitude of myometrium strips was exposed to different dosage of PKC-ε specific activator (Psi-RACK) in the organ bath experiments. **B:** mean values of AUC (area of under a curve) were recorded in myometrium strips opposed to different dosage of PKC-ε specific activator (Psi-RACK) in the organ bath experiments. **C:** blots show the expression of calponin and h1-calponin in the myometrium strips opposed to PKC-ε activator or inhibitor. **D:** columns from the ratio of phosphorylated h1 calponin to total h1 calponin in the myometrium strips opposed to PKC-ε activator or inhibitor. (0: control, I: Psi-RACK (PKC-ε activator) 10-9 mol/L, II: epsilon-V1-2(PKC-ε inhibitor) 10-9 mol/L, III: Psi-RACK 10-9 mol/L + epsilon-V1-2 10-9 mol/L). h1 Cap: h1 calponin; p- h1 Cap: phosphorylated h1 calponin. *: P < 0.05, compared with control.

The increased h1 calponin phosphorylation seen at the end of term could be due to many factors. To investigate whether PKC-ε directly regulates h1 calponin phosphorylation, we exposed mice myometrial strips in vitro to PKC-ε specific activator (Psi-RACK) [19]. Myometrial strips from late-pregnant (day 19, D19) mice were stimulated with the Psi-RACK after the spontaneous contraction of the strips had become stable. The concentration of Psi-RACK ranged from 10^-12^ mol/L to 10^-6^ mol/L. The changes of myometrium strips contractility was quantitated by integrating the force signal over a 15-min time period. Compared with control group without Psi-RACK, treatment with Psi-RACK in organ bath solution induced myometrium contraction in a dose-dependent manner (Figure 3A3B). The amplitude of myometrium strips contraction exposed to more than 10^-9^ mol/L Psi-RACK was significantly higher than that in control strips. To confirm that the contractile response was indeed due to PKC-ε activity, strips activated by 10^-9^ mol/L Psi-RACK and without were both treated with 10^-9^ mol/L epsilon-V1-2(PKC-ε specific inhibitor). Strips in Krebs solution without drugs acted as the sham-control. The results showed epsilon-V1-2 could reduce the amplitude of contraction of activated strips by Psi-RACK, but could not significantly reduce the contraction of strips which wasn't activated. We detected the expression level of h1 calponin and phospho-h1 calponin in myometrium strips with the method of western blot. We found that the ratio of phospho-h1 calponin to total h1 calponin got significantly increased in the strips exposed to 10^-9^ mol/L Psi-RACK as compared with control, but no difference among control groups, epsilon-V1-2 groups and Psi-RACK + epsilon-V1-2 groups. (Figure 3C3D).

## Discussion

As in all muscle tissue, the predominant proteins expressed in uterine smooth muscle are myosin and actin. In uterine smooth muscle, there is ~6-fold more actin than myosin [20]. Thin filaments (6-8 nm diameters) are polymers of globular monomeric actin. Thick filaments (15-18 nm diameters) are made up of myosin. In general, the actin and myosin filaments run in parallel and in the longitudinal dimension of the cell. All contractility is initiated by changes in the activity of, or interactions of, actin and myosin. In recent years, multitudes of signaling pathways have been suggested to regulate smooth muscle contractility; however, mechanisms that regulate the availability of actin to interact with myosin via the action of inhibitory actin binding proteins such as caldesmon (CaD) and possibly calponin (CaP) are strongly implicated in preterm labor[2]. During pregnancy, myometrial smooth muscle undergo profound and large changes, including disruption of the myofilaments or cytoskeleton. It is necessary to recover greater amounts of thin filament-associated proteins from nonpregnant tissues. Calponin may be expressed at levels reaching stoichiometric equivalence with actin, and has been proposed to be a load-bearing protein. H1 calponin is one of the thin filament proteins, which plays a role in the formation of cytoskeleton [21].

We have observed the protein expression of h1 calponin in the uterine of mice, which was detected with the method of Western Blot during different trimester of pregnancy. Here we demonstrated that h1 calponin protein level significantly increased in mice myometrium in late pregnancy and during labor compared with nonpregnant controls. The gain of h1 calponin may contribute to recover the cytoskeleton in the pregnant myometrium. The difference of the protein expression of h1 calponin not only demonstrated that the expression of h1 calponin was associated with the physiological state of mice but also suggested that the alternation of the h1 calponin during pregnancy laid the groundwork for regulating the contraction of mice uterine.

Reversible phosphorylation of proteins is an important regulatory mechanism that occurs in both prokaryotic and eukaryotic organisms. Many enzymes and receptors are switched "on" or "off" by phosphorylation and dephosphorylation. Protein phosphorylation is an omnipresent and important dynamic phenomenon in living systems that affects protein structure, protein-protein interactions and catalytic activity during physiological processes. The phospho-h1 calponin antibody used in this study is specific for h1 calponin phosphorylated at a PKC phosphorylation site, Ser175. PKC is a family including conventional isoforms(α, β, and γ), novel isoforms (δ, ε, η, and θ), atypical isoforms (i/λ and ξ) and PKD/μ [22]. Some studies indicate h1 calponin is the possible target of PKC-ε, and PKC-ε can interact with h1 calponin through the C-terminal repeats [13]. The PKC-ε is widely expressed throughout the body and has important roles in the function of the nervous [23], cardiac[24] and immune [25] systems. It is an attractive drug target for the treatment of several conditions such as inflammation [25], ischemia [26], addiction [27], pain [28], anxiety [29], and cancer [30]. Therefore, there is interest in discovering additional drug targets through identification of PKC-ε substrates and the signal pathways in which they participate.

In our experiments, it was been found that PKC-ε was activated with the use of its specific antibody in the western blot in labor, at the same time an increase in phosphorylation of h1 calponin at the Ser175 site was observed. The outcomes suggested that h1 calponin and its phosphorylation were helpful to or associated with labor. Meanwhile, we have designed the following organ bath experiments with the purpose of promoting the contraction of uterine myometrium at the time of Day 19 in pregnancy: 1. Muscle tension was recorded with isometric force transducers connected to a computer-controlled Gemini recorder (Chengdu Instruments, China) after the application of different dose of the activator of PKC-ε and of inhibitor of PKC-ε. 2. The levels of h1 calponin and phospho-h1 calponin were detected with the method of western blot spontaneously. The experimental results showed that the mean amplitude of the myometrium contractions and the ratio of phospho-h1 calponin/h1 calponin representing the degree of phosphorylation of the h1 calponin in the myometrial strips of mice were increased by exposing myometrial strips to Psi-RACK (the PKC-ε specific activator) more than 10^-9^ mol/L Psi-RACK, a PKC-ε specific activator, which can activate the PKC-ε to phosphorylated h1 calponin. Still, a positive correlation was found between the amplitude of the myometrium strips and the degree of h1 calponin phosphorylation. All these findings gave us suggestion that contractions of myometrium strips in labor mice were promoted by phospho-h1 calponin. Furthermore, another step was established to verify the effect of PKC-ε on the h1 Calponin: contractions of day 19 pregnant mice myometrium were recorded after adding some inhibitor of PKC-ε(epsilon-V1-2) followed by adding the PKC-ε activator(10^-9^ mol/L Psi-RACK) into the medium of organ bath. We found that epsilon-V1-2 could decrease the level of phospho-h1 calponin on stips activated by Psi-RACK, but had no significant effect on the contraction of non-activated strips. Epsilon-V1-2 is a 14- to 21-peptide sequence derived from the first variable region V1 of the regulatory domain of PKC-ε [31,32]. Its inhibitory activity is based on PKC-ε translocation and PKC-ε phosphorylation[33]. We conjectured that PKC-ε translocation and phosphorylation were not significantly happened in the strips without adding Psi-RACK. So epsilon-V1-2 had no significantly effect on the contraction of non-activated strips. The result of this step could imply that the phosphorylation of h1 Calponin was caused by the PKC-ε.

In vascular smooth muscle, through a PKC-ε-mediated h1 calponin phosphorylation, h1 calponin becomes disassociated from acto-myosin, allowing for cross-bridging, thus initiating smooth muscle contraction [12]. All findings in our studies strongly implicated PKC-ε-dependent h1 calponin phosphorylation have been linked to uterine smooth muscle contraction. Thus we postulate that PKC-ε-dependent h1 calponin phosphorylation may reverse the inhibition of actomyosin interactions by h1 calponin and may contribute to an increased uterine contractility during labor.

## Conclusions

In conclusion, we demonstrated that the PKC-ε activation, phosphorylation of h1 calponin at a PKC site reached the peak in myometrium in the mice in labor. Under the stimulation of the PKC-ε specific activator, the contractility of myometrium strips from pregnant mice was reinforced and the level of phosphorylated h1 calponin increased at the same time, which could be interrupted by the specific inhibitor of PKC-ε. These results constitute evidence for a role of the PKC-ε/h1 calponin pathway in the regulation of myometrial contractility and development of parturition.

## Competing interests

The authors declare that they have no competing interests.

## Authors' contributions

All authors participated in the design, interpretation of the studies, analysis of the data and review of the manuscript; LLS and ZY conducted the experiments and wrote the manuscript. All authors read and approved the final manuscript.
